# Supplementary material for: Photogrammetric approach to detect road pavement friction
Source: PLoS One. 2025 Feb 14;20(2):e0318188. doi: 10.1371/journal.pone.0318188 (PMC11828407; doi:10.1371/journal.pone.0318188)
Supplement: Attachment — Submitted filename: Reviewer Notes.pdf [file pone.0318188.s001.pdf]

After a comprehensive review of the research titled “Photogrammetric Approach to Detect Road Pavement Friction” submitted by the researchers and sent by your respected journal, the researcher reached the following:

1. In terms of the scientific idea, the research is good in the field of methods and presents a new idea in the working mechanism.
2. From a philosophical and literary perspective, the research contains very little of the proposition. Despite its importance in the research, it can be summarized as in a paragraph Current Knowledge line 72.
3. Are the twenty-three different approaches to evaluation based on standards or just studies? Please confirm line 43.
4. Recommendation to accept the research after making minor and pre-determined modifications.

With respect

Asst.Prof. Dr. Rana Amir Yousif
