# Supplementary material for: Photogrammetric approach to detect road pavement friction
Source: PLoS One. 2025 Feb 14;20(2):e0318188. doi: 10.1371/journal.pone.0318188 (PMC11828407; doi:10.1371/journal.pone.0318188)
Supplement: Attachment — Submitted filename: Response to Reviewers.docx [file pone.0318188.s002.docx]

**Reviewer #1:**

**1. In terms of the scientific idea, the research is good in the field of methods and presents a new idea in the working mechanism.**

- *Thank you!*

**2. From a philosophical and literary perspective, the research contains very little of the proposition. Despite its importance in the research, it can be summarized as in a paragraph Current Knowledge line 72.**

- *The main source for this research is Luhmann et al (Close-Range Photogrammetry and 3D Imaging) and the standards [5] ČSN EN 13036-4:2012 (73 6177) Road and airfield surface characteristics - Test methods - Part 4: Method for measurement of slip/skid resistance of a surface: The pendulum test. Most of the necessary information can be found in this literature. As the work is focused on an innovative method using a specific procedure, the availability of publications by other authors is very limited. From the point of view of the author's team, the use of existing references appears to be sufficient.*

**3. Are the twenty-three different approaches to evaluation based on standards or just studies? Please confirm line 43.**

- *The abstract has been modified to clarify that the 23 different surface evaluation approaches were determined based on the authors' experience during the measurements. In this context, standards were utilized to define the road surface samples.*

**4. Recommendation to accept the research after making minor and pre-determined modifications.
Thanks for the great research and new ideas.**

- *Thank you!*

**Reviewer #2:**

**1. problem statement not clear in the abstract**

- *The abstract has been modified to explain the aim of the thesis and its progress more clearly.*

**2. The conclusions are poor, need to rewrite**

- *Based on the submission guidelines of the journal, it was decided to combine the Discussion and Conclusion into one chapter. Only the Discussion is now included, where the authors believe that all the important conclusions of the paper are mentioned. At the same time, recommendations how to repeat the measurements have been added. At the same time, limiting factors that might limit the thesis have also been added.*

**3. Add recommendations**

- *If the authors have understood the question correctly, there is a section in the Discussion recommending how to make similar measurements.*

**4. Add ref to some equations mentioned in paper**

- *Has been added to the Manuscript.*

**5. Add limitation for this work**

- *Has been added to the Discussion.*

**6. the main gaps should mention and clarify according to previous related works.**

- *As the method of measurement is innovative, the main limitations cannot be sufficiently compared with previous work related to other studies. Comparisons have been made according to official standards dealing with pavement friction. These standards define how measurements can be made using the sand method and what results can be achieved (EN 13036-4:2012 (73 6177) Road and airfield surface characteristics - Test methods - Part 4: Method for measurement of slip/skid resistance of a surface: The pendulum test). Based on Luhmann et al (Close-Range Photogrammetry and 3D Imaging) it was possible to determine the necessary conditions for proper processing of the photogrammetric measurement. Then, the results show that the accuracy is sufficiently high and the measurement method can be considered as verified and valid.*
